# Behavioral, experiential, and physiological signatures of mind blanking

**DOI:** 10.1073/pnas.2510262122

**Published:** 2025-12-23

**Authors:** Esteban Munoz-Musat, Arthur Le Coz, Andrew W. Corcoran, Laouen Belloli, Lionel Naccache, Thomas Andrillon

**Affiliations:** ^a^Sorbonne Université, Institut du Cerveau-Paris Brain Institute (ICM, INSERM, CNRS, APHP), Hôpital de la Pitié Salpêtrière, Paris 75013, France; ^b^Université Paris-Cité/Service de Neurologie Cognitive, Hôpital Lariboisière-Fernand Widal (IHU reConnect), Paris 75010, France; ^c^Service des Pathologies du Sommeil, Hôpital Pitié Salpêtrière, Paris 75013, France; ^d^Monash Centre for Consciousness and Contemplative Studies, Faculty of Arts, Monash University, Melbourne 3168, VIC, Australia; ^e^Service de Neurophysiologie, Hôpital Pitié Salpêtrière, Paris 75013, France

**Keywords:** mind blanking, mind wandering, EEG, consciousness, attention

## Abstract

Does being awake necessarily mean being conscious of something? This study investigates the phenomenon of mind blanking (MB), characterized by an “emptiness of mind,” comparing it with mind wandering (MW) and on-task (ON) states. Using a sustained attention task and electroencephalogram monitoring, behavioral, and neurophysiological signatures of MB were examined in 62 participants. MB exhibited a specific pattern of behavioral lapses, as well as decreased fast oscillatory activity and complexity over posterior electrodes compared to MW. Functional connectivity analyses revealed decreased long-range interareal connectivity during MB, compared to both ON and MW states. Event-related potentials with source reconstruction and temporal decoding techniques indicated a significant disruption in visual processing during MB, starting from 200 ms post stimulus and echoing into the late-stage of visual processing, suggesting a disruption of conscious access to sensory information during MB. Electroencephalogram-derived markers allowed the prediction of mental states at the trial level, offering a finer view of conscious dynamics than subjective reports alone. Overall, these findings challenge the notion of the wakeful conscious mind as inherently content-oriented, suggesting that MB reflects genuine gaps in the stream of conscious thoughts, arising from disruptions in the generation or accessibility of thought content.

## Significance

Employing cutting-edge neurophysiological techniques on high-density electroencephalographic recordings, our study unveils unique neurophysiological markers of mind blanking (MB), a phenomenon characterized by lapses in conscious content amid the flow of wakeful consciousness. Distinguished from content-oriented states such as on-task and mind wandering, MB appears to be a distinct mental state. Furthermore, we demonstrate the feasibility of decoding consciousness dynamics solely from electroencephalogram features, transcending the limitations of intermittent subjective reports. Our findings thus not only provide a framework for investigating the stream of consciousness but also challenge the conventional notion that wakefulness invariably signifies being conscious of something.

*“Consciousness, then, does not appear to itself chopped up in bits. Such words as ‘chain’ or ‘train’ do not describe it fitly as it presents itself in the first instance. It is nothing jointed; it flows. A ‘river’ or a ‘stream’ are the metaphors by which it is most naturally described. In talking of it hereafter let us call it the stream of thought, of consciousness, or of subjective life”* (William James, 1890).

In the former quote, William James pointed to two very intuitive aspects of our conscious experience (1). First, consciousness seems continuous during wakefulness, without any “pauses” or breaks in the flow of contents of experience. Second, our conscious experience is dynamic and the origin of conscious content changes very frequently. Indeed, the stream of experiences can quickly shift between different external sources of information, but also turn inward toward internally generated task-unrelated thoughts, a phenomenon usually referred to as mind wandering (MW) (2, 3). Extensive research has shown that, in the brain, the transitions between task-related focus and MW involve modulations of activity of the Default-mode network (DMN) (4) as well as changes in cortical dynamics with modulations of alpha activity (5, 6) and increases in slower rhythms (7). However, a rigid separation between MW and task-focused states has been challenged by evidence emphasizing the role of context in shaping the neural correlates of MW (8, 9). Recent approaches, sometimes leveraging more naturalistic paradigms, focus instead on the experiential features of subjective experience: e.g., what kind of information is being processed and how (e.g., level of detail, engagement) (10, 11). For example, MW research has investigated the distinctions between different MW subtypes, stressing the importance of considering the meta-awareness (i.e., were participants aware of their MW) or voluntariness (i.e., did they mind-wander on purpose?) dimensions of MW (12). This perspective frames MW and task-focused states as points along a continuum within a multidimensional phenomenological space (13, 14).

More recently, a new “mind state” has been described, challenging the idea of a continuous stream of conscious contents during wakefulness: the state of mind blanking (MB). MB is described as a waking state that is either spontaneous or intentional, during which a subject does not report any mental content, but rather the feeling of an empty mind (15). Previous studies have shown that MB is reported about 14 to 18% of times during focused tasks (15, 16) and about 6% of times during resting (17). Furthermore, MB has been associated with specific behavioral outcomes, distinct from both MW and task-focused states (7). The exact nature of MB is still a matter of debate (18, 19) and only a few studies have attempted to investigate its neural correlates (20). Some have reported a widespread deactivation of thalamic and cortical brain regions during spontaneous MB (21) and a more focal deactivation of the superior and inferior frontal gyri and hippocampus during intentional MB, whereas the anterior cingulate cortex activation seemed to increase (22). Others have reported an increase of functional connectivity, as measured by phase coherence metrics, during MB (23). Finally, MB has been linked to the occurrence of sleep-like slow waves in scalp electroencephalogram (EEG) (7).

Two pressing questions regarding MB remain. First, it remains unclear whether MW and MB correspond indeed to different subjective and neurophysiological states (24, 25) or if they can be traced back to common underlying physiological causes expressed in varying degrees (26). Recent evidence suggests that both MW and MB could be explained by local sleep phenomena in the brain, but with different regional distributions (7). Other accounts suggest that MB could arise from a specific pattern of neural (de)activations or functional connectivity, distinguishable from both task-focused and MW states (21, 23). To better understand the true nature of MB in respect to both task-focused and MW states, the neural distinctions of these mind states, if any, need to be further investigated (20, 27).

Second, the place of MB in the hierarchy of consciousness states needs to be clarified. Traditionally, a clear distinction was made between states of unconsciousness (e.g., coma, deep N3 sleep) and states of consciousness (e.g., normal Wakefulness, REM sleep). States of consciousness (i.e., being conscious) classically imply the existence of conscious contents (i.e., being conscious of something) whereas states of unconsciousness imply the absence of such contents. Recent work suggests that the frontier between conscious and unconscious states is not as clear-cut. Indeed, conscious experiences (dreams) are often reported during consolidated non-REM sleep (28, 29); and transient behavioral as well as electrophysiological signs of conscious processing have been recently reported during sleep including N3 sleep (30–32). These results suggest an intriguing possibility: Could the reverse phenomenon—transient absence of conscious content during wakefulness—exist (27)? If so, could MB be a potential candidate for such a particular state?

Some theoretical accounts of consciousness align with the possibility of gaps in the stream of conscious contents. For example, the Global Neuronal Workspace Theory of Consciousness (GNWT) (33, 34) suggests that conscious access (and hence, reportability) to a given representation depends on the late, sustained, and global “ignition” of fronto-parietal associative areas. According to this view, consciousness is a discrete phenomenon, and windows of unconsciousness during Wakefulness could result from a gap between two ignitions. The Integration Information Theory (IIT) posits that consciousness would dissolve whenever the brain’s capacity to integrate information breaks down (35). Changes in cortical dynamics toward less integration may correspond to gaps in the stream of consciousness, much like the mirror phenomenon, local activations within sleep, is linked to dreaming (29). The parallel with sleep and dreaming research is particularly inspiring, since extensive research has shown that (macro) unconscious states (N3 sleep, deep anesthesia, coma, the vegetative state) are characterized by i) a decrease in neural complexity and fast neural oscillations (36–39); ii) a breakdown of information sharing between distant cortical areas (40–43), particularly in the delta and theta frequency bands (44, 45); iii) impaired neural responses and representations to external stimuli, particularly during the late-stage of information processing (>300 ms poststimulus) (38, 46–50). These previous studies provide candidate EEG markers to explore the signature of MB.

Here, we present compelling evidence that MB is a separate mental state, distinct from MW and task-focused states at the phenomenological, behavioral, and neurophysiological levels. To provide an exhaustive characterization of the neurophysiological fingerprint of MB, we relied on previous work exploring consciousness states and contents (51). In particular, we capitalized on the use of markers tracking both background neural dynamics (complexity, information sharing) and the processing of external information (ERPs, temporal decoding) in the sensor and source space. Based on these signatures, we argue that MB could represent a failure of conscious access mechanisms, resulting in a genuine gap in the stream of conscious contents.

## Results

### Different Behavioral Signatures between MB and MW.

**Fig. 1.**
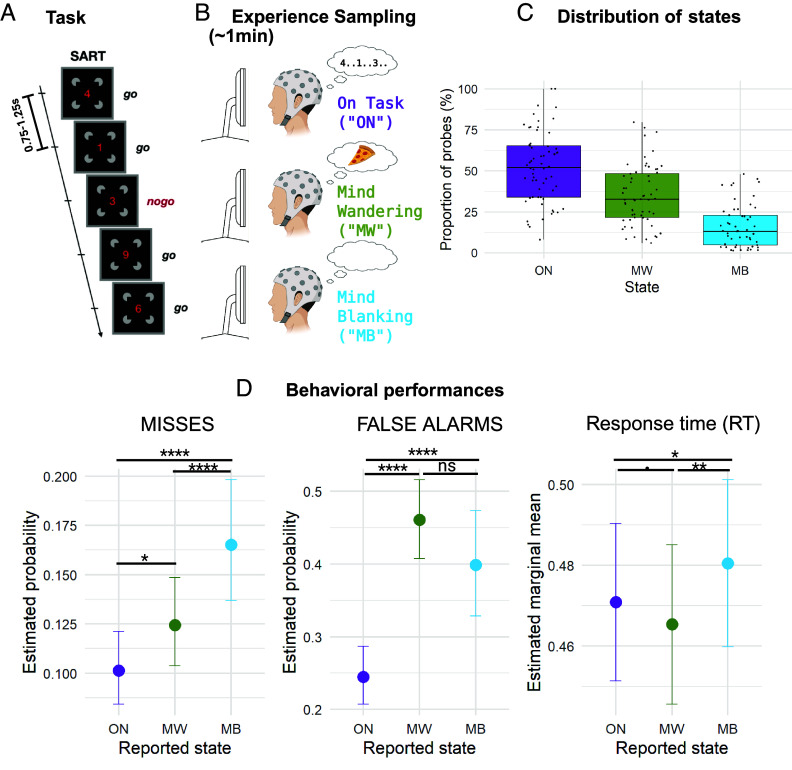
Experimental paradigm and behavioral results. (*A* and *B*) Experimental paradigm. 62 healthy participants performed a SART, with digits and faces as stimuli, while hdEEG was recorded continuously. After each stimulus presentation, participants had to press a button (Go trials), or refrain from doing so, whenever a 3 or a smiling face was presented (No Go trials; 1 out of 9). SOA varied randomly (750 to 1,250 ms) with a null ISI (*A*). Participants’ mental state was probed at random intervals (every 40 to 70 s, uniform random jitter). They had to report their attentional focus by selecting one of four options: 1) task-focused (ON), 2) off-task (MW), 3) MB (*B*), and 4) don’t remember. (*C*) Proportion of probes in function of the reported mind state. Proportions were computed at subject level (individual dots) and the distributions at group level are represented by a boxplot [the boundaries of the boxes represent first and third quartiles (Q1 and Q3 respectively), mid-line represents the medium and the whiskers depict Q1-1.5*IQR and Q3*1.5*IQR]. (*D*) Behavioral performances in function of the reported mind state. Misses, false alarms (FA), and response times (RTs) were computed for the trials between the 5 s before probe-onsets. (Binomial) linear mixed models were computed with *mind state* as the main explanatory factor, and subject ID/dataset as a random intercepts. We found fine-grained modulations of performance in function of mind state, with distinct behavioral profiles for MW (faster RTs) and MB (slower RTs, more misses). The statistical bars and stars represent the pairwise comparisons between mind states (FDR corrected). The detailed statistical results can be found in *SI Appendix*, Table S1. ****: FDR corrected *P*-value < 0.0001, **: FDR corrected *P*-value < 0.01, *: FDR corrected *P*-value < 0.05,.: FDR corrected *P*-value < 0.1, n.s.: FDR corrected *P*-value > 0.1.

In these two studies, 62 healthy participants performed a modified sustained attention to response task (SART), with digits and faces as stimuli presented in separate blocks, while high-density electroencephalogram (hdEEG) was recorded continuously. After each stimulus presentation, participants had to press a button (Go trials), or refrain from doing so whenever a “3” or a smiling face was presented (No Go trials; 1 out of 9). Stimulus onset asynchrony (SOA) varied randomly (from 750 to 1,250 ms) with a null interstimulus interval, and participants’ mental state was probed at random intervals (every 40 to 70 s, uniform random jitter). Participants were instructed to report their attentional focus “just before the interruption” by selecting one of four options: 1) “task-focused” (ON), 2) “off-task” (MW), 3) MB, or 4) “don’t remember.” Since the fourth option accounted for only 2.6% of all probes (i.e., less than two probes per participant on average) and given that previous studies did not consistently distinguish between these options (52), we merged third and fourth options as MB in all analyses (Fig. 1 *A* and *B*).

Participants performed this task with a high accuracy [mean accuracy of 86.5 ± 0.5% (SEM)] and with a relatively low rate of Misses (i.e., errors in Go trials, 10.4 ± 0.5%). Errors in No Go trials (FA) were frequent (36.7 ± 1.5%). At the subjective level, participants reported being focused on the task (ON) in only 52 ± 2.7% of the probes and otherwise reported MW (35 ± 2.3%) or MB (16 ± 1.8%) (Fig. 1*C*). The distribution of the ON, MW, and MB mind states was not uniform across participants (Fig. 1*C*), with large interindividual differences: In particular, six participants (9.6%) did not report any MB experience but reported MW experience, two participants (3.2%) reported no MB and no MW and no participants reported MB without MW.

In order to detect fine-grained modulations of behavioral performance according to the participant’s mind state, we labeled all trials presented up to 5 s before each probe as corresponding to the probed state (e.g., after an “ON” response, all previous trials that occurred during the previous 5 s were labeled as ON trials). As previously reported in a smaller subgroup of participants (40% of the present cohort) (7), we found a significant main effect of *mind state* on FA, misses, and RTs (*SI Appendix*, Table S1). Crucially, pairwise comparisons between mind states revealed different behavioral profiles for MW and MB: While both were characterized by more FA and misses than during ON, FA were equally frequent during MW and MB (with a nonsignificant trend for more FA during MW trials) and misses were more frequent during MB (i.e. MB>MW>ON) (*SI Appendix*, Table S1 and Fig. 1*D*). RTs were slower for MB (compared to both ON and MW), and faster for MW (compared to MB and ON; significant difference for MW vs. MB and statistical trend (FDR corrected p-value 0.1) for MW vs. ON) (*SI Appendix*, Table S1 and Fig. 1*D*). In sum, while MW seemed characterized by an “impulsive profile” (more FA and faster RTs), MB presented with an “absent profile” (more misses and slower RTs), which confirms our previous findings obtained in one of the two datasets.

### “Front-Back” Dissociation between MB and MW.

We next investigated neurophysiological correlates of the mind states (ON, MW, and MB) from EEG recorded during the 5 s preceding each probe onset. We computed three classes of neural markers: 1) spectral markers [normalized power spectral densities (PSD) in the delta, theta, alpha, beta, and gamma bands]; 2) complexity markers (38, 53) [Kolmogorov complexity (KC) and sample entropy (SE)] and 3) connectivity markers [*weighted symbolic mutual information* (44) (wSMI) in the delta, theta, and alpha bands]. These markers (and other similar measures) have been previously shown to track modifications of cognitive and consciousness state in a large range of conditions, including disorders of consciousness (DoC) (38, 39, 44), sleep (45, 54, 55), hypnosis (56), and meditation (57).

All studied neural markers were modulated by the participants’ mind state. A significant main effect of *mind state* and of *electrode location* was found for the wSMI in the delta and theta frequency bands, and a significant effect of these two main factors (mind state, electrode location) and their interaction was found for all other markers (wSMI alpha; normalized PSD delta, theta, alpha, beta, gamma; SE and KC) using linear mixed models with mind state, electrode location and their interaction as main factors. Subject ID and dataset were introduced as random intercepts (*SI Appendix*, Table S2).

**Fig. 2.**
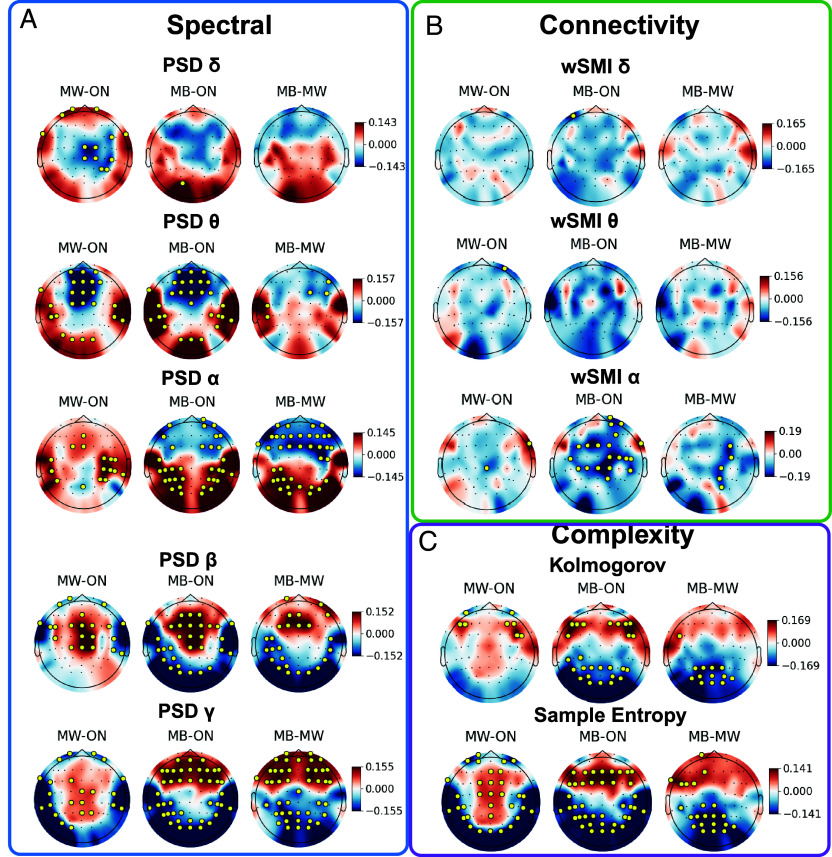
EEG-based neurophysiological markers differentiate MB from ON and MW. Spectral (*A*), connectivity (*B*), and complexity (*C*) results. Each subplot represents the pairwise statistical contrast between two states (*Left*: MW–ON; *Middle*: MB–ON; and *Right*: MB–MW), at sensor level (topographical representation of the scalp). Model estimates for the contrast between states were computed for each electrode, and locations with statistically significant differences (FDR corrected *P*-value < 0.05) are depicted with a golden circle. We observed a front-back dissociation between MW and MB for spectral and complexity measures, and a progressive breakdown of functional connectivity going from ON to MB (ON>MW>MB). The ANOVA tables summarizing the main factor’s effects (linear mixed models with mind state, electrode/connection and their interaction as main factors, and subject ID/dataset as a random intercepts) for each EEG metric can be found in *SI Appendix*, Table S2.

Spectral and complexity measures revealed different profiles for MW and MB: compared to ON, MW was associated with both an increase in fast oscillatory activity and complexity over central electrodes, and with a decrease of these metrics over fronto-polar and posterior electrodes. As for MB, compared to ON, it was associated with an increase in fast oscillatory activity and complexity over frontal and fronto-central electrodes, and with a decrease in these metrics over centro-posterior and posterior electrodes. The direct contrast between MB and MW revealed a front vs. back dissociation: While frontal and fronto-central electrodes showed faster oscillatory activity and higher complexity in MB than in MW, centro-posterior and posterior electrodes showed an opposite difference (Fig. 2 *A* and *C*).

Mind state can be modulated by arousal, with MW and MB frequency increasing in moments of hypovigilance (7, 58). Therefore, some of the observed changes in neural markers could be explained by a decrease in alertness. To ensure that our results could not be *exclusively* explained by this arousal factor, we conducted a verification analysis, by including in our statistical model the vigilance score as a covariate (4-point scale, 1 = Extremely Sleepy, 4 = Extremely Alert), as well as the interaction between electrode location and vigilance score. The observed results for spectral and complexity measures were nearly identical to those previously presented, with the persistence of a clear front-back dissociation between MW and MB. See *SI Appendix*, Fig. S1 *A* and *C*.

### Breakdown of Long-Range Information Sharing During MB.

**Fig. 3.**
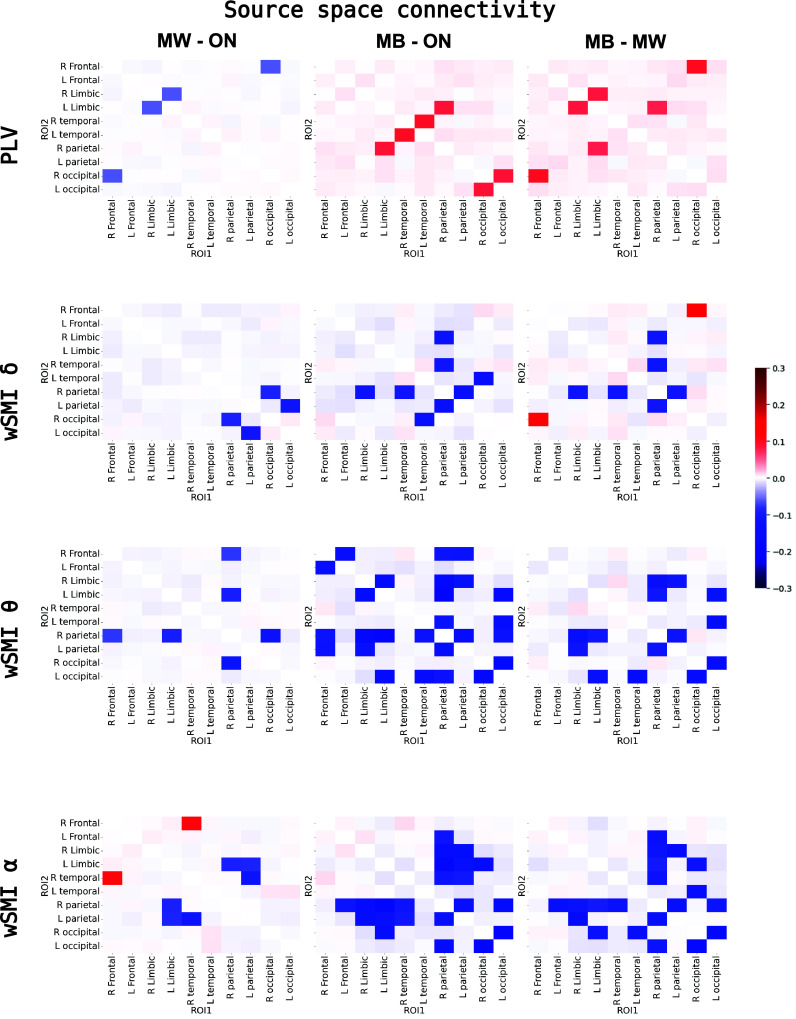
Increased phase synchrony with reduced information sharing during MB. The PLV (*Top*) and the wSMI in different frequency bands were computed at the source level. Each square-matrix represents the contrast in connectivity, computed in source space, between two mind states, for each pair of regions of interest (ROIs). Only significantly different connections (FDR corrected *P*-value < 0.05) are highlighted, the other ones are masked. ANOVA tables for the statistical models can be found in *SI Appendix*, Table S3. We observed a dissociation between the PLV and wSMI, with increased PLV (phase synchrony) and decreased wSMI (information sharing) between distant cortical areas (in particular fronto-parietal) during MB, as compared to ON and MW. δ: delta band; θ: theta band; α: alpha band wSMI: weighted symbolic mutual information PLV: Phase Locking Value R: right; L: left.

Functional connectivity analyses revealed a significant modulation by *mind state* of the wSMI metric in frequency bands of interest (delta, theta, and alpha) (*SI Appendix*, Table S2). Post hoc topographical analyses in sensor space revealed a progressive breakdown of connectivity from ON to MW, and then from MW to MB, particularly in the alpha band (Fig. 2*B*). As for spectral and complexity measures, the inclusion of vigilance score as a covariate in statistical models resulted in very similar results (*SI Appendix*, Fig. S1*B*). To better characterize these connectivity modulations in function of mind state, we reconstructed the cortical sources of the EEG signal (N = 68 cortical sources) at the trial level and computed the information metric wSMI (at different frequency bands) between each pair of sources (n = 2,278 connections). These sources were further grouped in 10 pairs (right and left) of 5 regions of interest (ROIs), according to the Desikan-Killiany atlas (frontal, limbic, temporal, parietal, and occipital), obtaining thus 45 averaged connections between ROIs. Since previous studies reported modifications of coherence-based metrics during MB, we also computed in source space the phase locking value (PLV), for comparison. The statistical analysis revealed a main effect of *mind state* for all computed connectivity metrics (linear mixed model with mind state, connection and their interaction as main factors; subject ID and dataset were introduced as random intercepts; see *SI Appendix*, Table S3). As reported in a previous study (23), the coherence-based metric PLV showed an increase in interareal connectivity during MB, compared to both MW and ON states (Fig. 3, *Top* panel). By contrast, the information-based metric wSMI showed the reverse pattern: For all computed frequency bands (delta, theta, and alpha), a significant reduction in interareal connectivity was observed during MB, compared to both ON and MW (except for the connectivity in the delta band between right frontal and right occipital areas, which increased) (Fig. 3). This disruption of interareal connectivity during MB concerned mainly parietal areas, and in particular fronto-parietal connectivity.

The inclusion of the vigilance score in the statistical models led to results largely consistent with those previously presented for the PLV and the wSMI in the theta and alpha bands; for the wSMI in the delta band, the results were more nuanced, revealing both increases and decreases of long-range connectivity during MB (*SI Appendix*, Fig. S2).

As it will be discussed later, the contrast between the PLV and the wSMI results could reflect the relative implication of linear vs. nonlinear interactions between cortical regions during these different mind states, nonlinear interactions being best captured by the wSMI (*Discussion*).

As an interim conclusion, these neurophysiological findings confirmed and extended our behavioral results, by showing that MB is associated with a specific brain state distinct from MW. In addition, the significant disruption of long-range connectivity revealed by the wSMI metric during MB suggests a marked impairment of information sharing between cortical areas during this mind state, as systematically observed during unconscious states.

### Disruption of the Brain Processing of External Stimuli During MB.

The processing of external information is very dependent upon the background brain activity and associated mind state, and foremost the state of consciousness. Previous research has shown that early and intermediate cortical processing is preserved in classically unconscious states such as non-REM sleep (49, 59–63) or coma (64–66) (having a prognostic value in this last case). By contrast, late cortical processing seems to be associated with conscious states (30, 46, 47, 49) and conscious processing of external information (67–70). With this in mind, we decided to probe the differential neural fate of the presented visual stimuli according to the reported mind state.

**Fig. 4.**
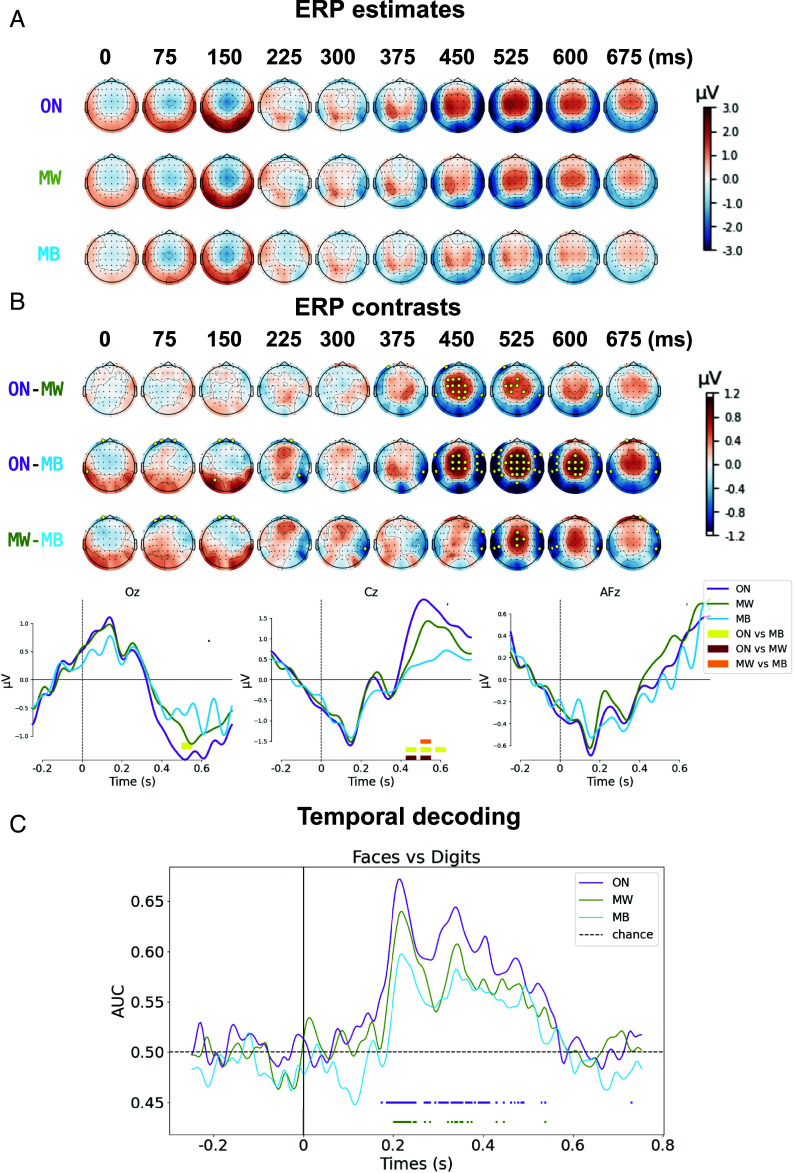
Stimulus-induced activity analyses. (*A* and *B*) Topographical scalp representation of the ERP estimates for each mind state (*A*) and ERP contrasts between mind states (*B*), derived from the statistical model (single-trial multilevel analysis with mind state, EEG channel and their interaction as main factors, and subject ID/dataset as random intercepts). Marked electrodes in *B* (golden circles) are the ones presenting a statistically significant difference between conditions (FDR corrected *P*-value < 0.05). *B*, *Bottom* presents the grand-average ERP time-series for each mind state (ON: violet; MW: green; MB: blue) for three electrodes of interest (Oz, Cz, and AFz). Time-points with statistically significant differences between conditions (FDR corrected *P*-value < 0.05) are highlighted (ON vs. MW: brown; ON vs. MB: yellow; MW vs. MB: orange). Given that trials follow one another in close succession, it is normal for the baseline activity to be influenced by the activity of the previous trials. (*C*) Temporal decoding of stimulus category. A linear classifier was trained and then tested (via cross validation) at each time point to distinguish stimulus category (faces vs. digits), allowing the tracking of the temporal dynamics of neural representations, independently for each mind state. Note that the number of trials were identical between mind states for this analysis. Classifiers’ performances were compared to chance-level performance (*Methods*), independently for each mind state, and statistically significant time-points were represented by colored horizontal bars. While both early (<300 ms) and late (>300 ms) time-points with significant decoding were observed for ON and MW, no significant decoding was observed for MB trials.

First, we conducted a single-trial multilevel analysis of event-related potentials (ERPs) in sensor space (*SI Appendix*, Table S4 for the results of the statistical model). Early processing of visual stimuli was extremely similar across ON, MW, and MB mind states. In particular, a similar posterior positivity peaking around 100 to 150 ms after stimulus presentation, and corresponding to the P1 component (71), was present in all three conditions (Fig. 4*A*). Visually, this P1 component seemed more pronounced in ON and MW states than in MB, but very few electrodes showed a significant effect (≤3). No significant differences were observed between MW and ON during the early time-window (Fig. 4*B*). Crucially, stimulus processing diverged massively as a function of mind state during the late time-window (>350 ms). In ON trials, a classical P3b component (central positivity) spanned roughly from 400 to 650 ms after stimulus onset. In MW trials, this P3b pattern was still observable, but with a longer latency, a reduced maximal amplitude and a shorter duration. Finally, in this late time-window, no clear P3b response was observable in MB trials; amplitudes of MB ERPs were significantly decreased as compared to ON and MW, with only a reduced and left-lateralized response, possibly related to motor responses (Fig. 4 *A* and *B*). As a control, we also conducted the previously described analysis independently for each stimulus type (faces vs. digits). ERP profiles show differences in the timing of early and late components yet, for digits and faces, MB trials were characterized by a disruption of late components compared to ON trials (*SI Appendix*, Fig. S3).

While univariate methods such as ERPs provide with important information about the amplitude and timing of cortical activations in response to external stimuli, multivariate decoding methods allow for a more subtle analysis, and provide information at the level of neural representations. We wanted to answer the following question: Does brain activity encode relevant information about the presented stimuli, and how these neural representations vary as a function of the reported mind state? To do so, we tested whether we could decode stimulus type (faces vs. digits) from brain responses using multivariate pattern analysis (MPVA), with the temporal decoding method (48, 72). Briefly, this type of analysis consists in using a subset of the data to train a linear classifier at each time-point to differentiate trials where a *Face* was presented from trials where a *Digit* was presented, and then testing its performance, independently at each time-point, in a different subset of the data. We ensured a comparable number of trials between mind states by randomly subsampling trials from each mind state. Then, we applied the temporal decoding method independently for each mind state, and statistically compared the obtained performance scores (ROC AUC) with a dummy random distribution centered around 0.5 (chance-level performance). Results can be found in Fig. 4*C*. For ON trials, we found several time-windows with significant decoding compared to chance-level performance. The first one, around 200 ms, coincided with a sharp decoding peak (AUC > 0.65). The other ones, spanning from 250 ms to 580 ms, corresponded to a sustained period of decoding. For MW trials, we found a similar initial peak around 200 ms; however, later time-windows with significant decoding were sparser, with a period around 350 ms, and then a few significant time-points after 400 ms. Crucially, we did not observe any statistically significant decoding for MB trials.

**Fig. 5.**
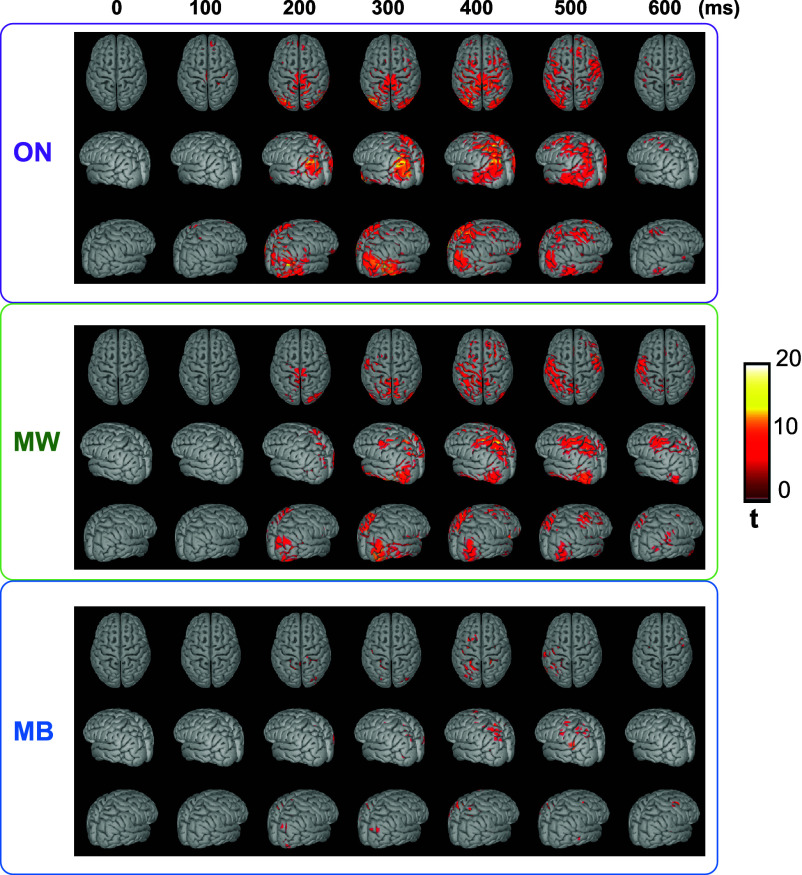
Source reconstruction of stimulus-induced activity. Source reconstruction at trial level was performed independently for each mind state, and a i test against the baseline (−0.25 to 0 s relative to stimulus onset) was performed. Only statistically significant modulations of activity compared to the baseline (FDR corrected *P*-value < 0.05, corrected for multiple comparisons across time, space, and frequencies) are highlighted. While significant activations of visual streams (dorsal and ventral) were observable for both ON and MW conditions, starting from 200 ms poststimulus, this was not observed during MB trials.

Given these results, we reconstructed the cortical sources of the EEG signals at trial level, and compared the poststimulus activations (time-window: 0 to 700 ms) to the activations during the baseline period (−250 to 0 ms). For both ON and MW trials, we observed a significant activation of bilateral occipital visual regions (around 200 to 300 ms poststimulus), followed by an activation of regions along the ventral and dorsal visual streams (300 to 600 ms), reaching some frontal areas (around 500 ms post stimulus). Responses in MW trials were smaller, delayed, and less spatially extended compared to ON. Importantly, for MB trials, we observed only sparse activations, mostly left-sided (contralateral to motor responses), with no clear involvement of the dorsal or ventral visual streams (Fig. 5).

In sum, while the essential processing steps of visual information seem preserved during MW compared to ON-task, these processes appear significantly disrupted during MB, particularly the late responses usually associated with conscious access.

### Trial-by-Trial Prediction of Mind State Based on EEG Neural Features.

**Fig. 6.**
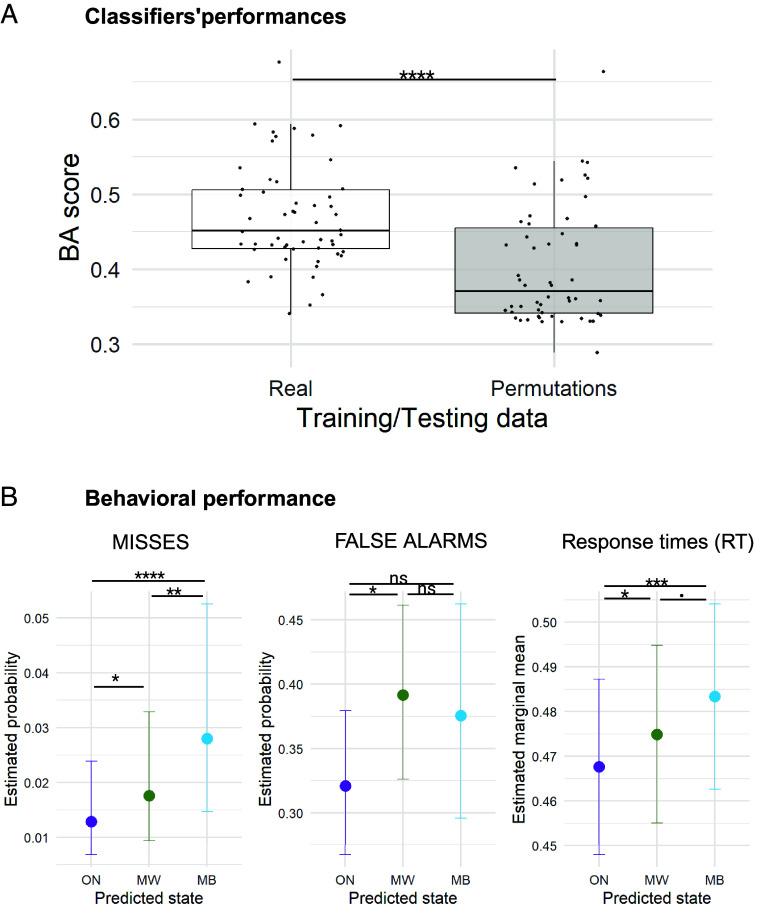
Trial-by-trial prediction of mind state using a multivariate combination of neural markers. (*A*) Distribution of performances (balanced accuracy) of subject-level classifiers, both for true data and permuted data (mean score of 500 labels permutations). The classifier was trained and tested by cross-validation using labeled trials (<5 s before probe onsets). Spectral, complexity, connectivity, and ERP markers were used as raw features, followed by a dimensionality reduction by (nonlinear) PCA. Only subjects presenting with the three mind states were included in this analysis. (*B*) Behavioral markers as a function of the predicted mind state in nonlabeled trials (>5 s before probe onsets). (Binomial) linear mixed models were computed with predicted mind state as the main explanatory factor, and subject ID/dataset as random intercepts. Statistical bars represent pairwise statistical comparisons between states. We found very similar behavioral patterns for predicted trials as compared to reported (labeled) trials. The detailed statistical results can be found in *SI Appendix*, Table S6. ****: FDR corrected *P*-value < 0.0001, ***: FDR corrected *P*-value < 0.001, **: FDR corrected *P*-value < 0.01, *: FDR corrected *P*-value < 0.05,.: FDR corrected *P*-value < 0.1, n.s.: FDR corrected *P*-value > 0.1.

The exploration of the dynamics of consciousness is limited by the reliance on the discrete sampling of experience through mind probes and subjective reports. To circumvent this snapshot approach, we set out to predict participants’ mind states on a trial-by-trial basis using neural features. We gathered all the previously presented EEG markers from stimulus-centered epochs (−0.25 to 0.75 s relative to stimulus onset), for all trials. We then trained and cross-validated, independently for each subject, machine learning classifiers (random forest), using exclusively trials within the 5 s preceding probe onsets (since we can label these trials as “ground truth” based on subjective reports). Importantly, during the cross-validation procedure, we grouped trials by experimental block (six blocks for each participant) to avoid overfitting due to temporal proximity between trials. In multiclass classification (ON vs. MW vs. MB), the median classifier’s balanced accuracy score across participants was of 46%, significantly above chance-level performance [median chance-level (500 permutations): 37%, FDR corrected *P*-value = 9 × 10^−6^, Wilcoxon rank-sum test] (Fig. 6*A* and *SI Appendix*, Tables S5 and S6). Classifiers’ performances varied sharply between participants with classification scores above 60% for some participants, while for other participants the classifier presented with chance-level performance.

We then used our trained classifiers to estimate the mind state in nonlabeled trials (>5 s before probe onsets). To estimate the reliability of these single-trial predictions, we computed metrics of behavioral performance in these trials for each predicted state ($\hat{\mathrm{ON}}$, $\hat{\mathrm{MW}}$, $\hat{\mathrm{MB}}$). Indeed, as shown previously, ON, MW, and MB have different behavioral signatures so we can use behavior to check whether these signatures are consistent with our predictions. And indeed, behavioral patterns of predicted states were very similar to the corresponding labeled states: We observed more misses in (predicted) $\hat{\mathrm{MB}}$ trials than in $\hat{\mathrm{ON}}$ and $\hat{\mathrm{MW}}$ trials, as well as longer RTs in $\hat{\mathrm{MB}}$ as compared to $\hat{\mathrm{MW}}$ and $\hat{\mathrm{ON}}$ trials (*SI Appendix*, Table S7 and Fig. 6*B*). We also observed more FA in $\hat{\mathrm{MW}}$ compared to $\hat{\mathrm{ON}}$ trials but RT were significantly slower and not faster (Fig. 1). This pattern of results suggest that our classifiers were able to retrieve the behavioral signatures on ON, MW, and MB states for unprobed trials.

## Discussion

### MB Corresponds to a Specific Brain State, Different from MW.

In this study replicating and extending a previously published study (7), we examined the behavioral and neural correlates of MB, compared to those of MW and ON states. During a sustained attention task, we confirmed our previous findings (7) showing that MB corresponds to a distinct mental state, characterized by a specific behavioral profile and by specific neural signatures. At a behavioral level, MB was characterized by response slowing and higher rate of misses, compatible with participants’ subjective report of having been “absent minded” during those moments. This behavioral profile was different from the one observed during MW that was characterized by an acceleration of responses, suggestive of an “impulsive” pattern of behavior. These differences in behavioral outcomes between MW and MB were previously reported in different tasks (e.g., reading) (73) or even task-free resting state (RT computed on probe responses) (58). At the neural level, both state markers (spectral, complexity, and connectivity measures) and brain responses to external stimuli differentiate MB from MW and ON states. This complements previous findings in fMRI associating spontaneous MB with a pattern of whole-brain hyperconnectivity and deactivation (21, 23). A multivariate combination of these neural metrics allowed for higher than chance prediction of mind state on a trial-by-trial basis, further supporting the idea that MB, MW, and ON states correspond to different mental and neurophysiological states. Taken together, these multimodal results reaffirm the specificity of MB as a distinct mental state (20).

These results face the intrinsic limitations of the validity and reliability of self-reports during experience sampling (74). First, nine participants never reported any instance of MB, raising the possibility that the occurrence of MB is trait-dependent, an idea supported by its higher prevalence in individuals with attention-deficit hyperactivity disorder (ADHD) (52, 75, 76). It is also possible that individuals show different response biases in reporting MB. Second, since we used a probe-catching method where subjective reports were assessed every 1 min or so, we cannot exclude the possibility of mental contents/states happening during the interprobe intervals that participants could not report. Third, the experience sampling method does not allow to determine precisely the time window that corresponds to a given mind state. As a first step toward addressing this limitation in the future, we tried to predict the participant’s mind state without reports using multivariate classifiers trained with neural metrics during those trials. Finally, our criterion for considering trials as belonging to a particular mind state (5 s) was motivated by technical reasons, and not hypothesis or data-driven.

### Front vs. Back Dissociation between MB and MW.

We found that spectral and complexity markers display opposite topographical profiles for MW and MB: Whereas MB was characterized by an increase of both complexity and fast oscillatory activity over frontal electrodes (compared to MW), it was also characterized by a decrease in these same metrics over posterior electrodes. These results are in line with previous analyses in a subset of our dataset (40% of the current data) (7), that found evidence for sleep-like slow-waves in both MW and MB, but with different regional localizations: While MW was associated with the presence of wake slow-waves over frontal scalp regions, MB was associated with their extension to posterior regions. Two previous fMRI studies showed that spontaneous MB is associated with an extensive cortical and thalamic deactivation as well as a widespread positive phase-coupling between brain regions (21, 23). Interestingly, chemogenetic inhibition of the mouse prefrontal cortex can result in i) a decrease in neuronal firing, ii) the presence of slow oscillations, iii) an increase in functional connectivity (77), which suggests that the different neural correlates of MB (slow waves, deactivation, and hyperconnectivity) could reflect the same neural mechanisms.

These results contrast with another study in fMRI focusing this time on voluntary MB, which showed its association with a deactivation of lateral prefrontal regions and hippocampus, but an activation of the anterior cingulate cortex (22). While our spectral and complexity measures were computed in sensor space, and therefore lack precise anatomical information, the observed increase in fast oscillatory activity and neural complexity over centro-anterior electrodes during MB could also be related to ACC activation. These findings are also in line with studies contrasting episodes of MW in which participants are aware or unaware of their own MW. When caught unaware, participants exhibited more activation in frontal areas (dorsal anterior cingulate cortex and ventral pre frontal cortex) (78), which could be related to the increase in fast oscillations we observed in MB, possibly suggesting a continuum between MB and unaware MW. Overall these results highlight the necessity to refine the phenomenological characterization of MB in order to better apprehend its neural correlates (18, 19). It further enriches the literature on spontaneous thoughts and experiences, which previously focused on MW but stressed the importance of considering phenomenal dimensions of MW such as meta-awareness, voluntariness, or emotional valence. With MB, we emphasize that the continuous presence of experiential contents should not be taken for granted.

### Increased Phase Synchrony with Decreased Information Sharing between Distant Brain Regions During MB.

To date, previous studies have reported mixed results regarding functional connectivity during MB. A previous study using fMRI (22) revealed lower functional connectivity between the default mode network and frontal, visual, and salience networks during voluntary MB as compared to MW. By contrast, using a coherence-based measure, a recent study (23) found a pattern of global positive connectivity during spontaneous MB. Beyond potential differences between voluntary and spontaneous MB, it is important to consider that functional connectivity results depend very much on the type of measure being computed. In the present study, we computed two different metrics: the PLV (79), a classical coherence-based measure that captures strictly linear correlations, and the wSMI, an information theory based measure which favors nonlinear correlations over purely linear ones (44). While the PLV showed an increase of interareal connectivity in MB as compared to both ON and MW (in line with the previously mentioned study), the wSMI revealed the inverse pattern, with reduced interareal (and more specifically, fronto-parietal) connectivity. Dissociations between coherence-based measures and the wSMI have already been reported, for example when contrasting Wakefulness and N3 sleep, where the whole-brain wSMI was significantly decreased in N3 sleep compared to wakefulness, whereas the wPLI (weighted Phase Lag Index) was not (80). Our results could reflect a dissociation between increased phase-synchrony with decreased information sharing in MB, which could be caused by the occurrence of synchronous episodes of slow waves and neural silencing over associative cortices (81, 82). More frequent regional sleep-like slow waves in MB could explain the increase in PLV as these events would realign the phase of EEG signals across sources (7, 83). Several lines of evidence suggest that MB may mark the beginning of the transition into sleep. Sleep onset is not a discrete event but a gradual and multifaceted process (84). Consistent with this view, MB is associated with behavioral slowing (increased misses and longer reaction times), alterations in brain connectivity and fronto-parietal sensory processing, and a shift toward sleep-like neural activity (7). MB also increases with sleep deprivation (58). However, it is important to emphasize that participants remained responsive to the SART constant visual stimulation, with fewer than 20% misses in the MB condition. Thus, if MB reflects a state closer to sleep, it still occurs within a globally wakeful state from both behavioral and physiological perspectives (85). Last, this model stresses the influence of episodes of hypoarousal in the occurrence of MB but does not exclude that MB could also occur in states of hyperarousal as suggested elsewhere (58).

### Disrupted Neural Representations of External Stimuli During MB.

Stimulus-induced activity analyses (ERPs, source reconstruction, and temporal decoding) revealed similar patterns of processing of task stimuli during ON-task and MW states, significantly different from those observed during MB.

The most obvious difference between mind states concerned the P3b component. A P3b response (central positivity between 400 and 600 ms poststimulus) was clearly present during ON-task, was reduced but still observable during MW and seemed to lack during MB trials. While the neural correlates and signatures of the conscious processing of external information remain highly debated (86, 87), previous studies (46, 68, 69) have proposed the P3b component as an EEG signature of conscious access. This interpretation remains debated, since some authors have claimed that the P3b relates more to postperceptual processes associated with (external) report than to conscious awareness per se (88, 89). The reduction of the P3b observed in MW (shorter duration and decreased amplitude compared to ON-task) is in line with previous findings showing the impact of top–down attention on the P3b (90). This reduction suggests the superposition of stimulus-unrelated processes during the time-window of the P3b response during MW. This last interpretation is in agreement with a recent study (67), which demonstrated that the P3b can be modulated by participants’ attentional focus but conscious perception is always associated with a P3b-like activation of a broad set of associative cortical areas (frontal, parietal, and temporal) even in the absence of report. When participants were paying attention and reported on the stimuli, additional cortical areas becoming involved during the same time window lead to a full-fledged P3b. Our source localization results align with this model with a similar activation of the dorsal and ventral visual streams during the late time-window for both ON and MW conditions, reaching some frontal areas in both conditions, but with a less spatially extended response during MW. Crucially, MB showed here a very different ERP signature at the sensor and source level. The relative absence of a P3b response as well as the absence of clear activation of the ventral/dorsal visual streams strongly suggests the absence of conscious access to external visual stimuli in MB trials in contrast with both ON and MW trials.

We also observed differences at the level of early visual processing between the different mind states. While no major differences were observable in sensor-space ERPs during the early time-window (<300 ms poststimulus), our temporal decoding analysis revealed the emergence of distinct visual neural representations around 150 to 200 ms poststimulus for both ON and MW trials (significant decoding against chance-level performance of stimulus category), while this was not the case for MB trials (despite the fact that the number of trials were balanced across conditions). The absence of a significant encoding of visual stimuli over occipital areas around 200 ms could suggest a disruption of the actualization of visual representations during MB. This aligns with the pattern of global cortical deactivations reported during MB (21). It further suggests that the lack of late potentials observed during MB could be due to a weaker or absent sensory activation, failing to ignite the cascade of activations observed in ON and MW, albeit with decreased amplitude for the latter.

### Probing Mind State without External Reports.

The stream of conscious experiences is private and probing for its contents and dynamic is only possible with a sparse and disruptive sampling approach. We attempted here to bypass these reports by leveraging our correlates of MW and MB. We showed first that we could predict above chance the mind state category of trials just before a probe (using the mind state reported following the probe as a ground truth) using a multivariate combination of different neural markers. Second, we applied the same algorithm to predict mind states for trials away from the probes, so during moments where we do not have participant’s mind state reports. We could retrieve the behavioral signatures of these states, which suggests that this MVPA approach partially captures a similar dynamics of conscious experience as evidenced in trials in which participants reported on their mental state. While the accuracy of our classifier approach remains very limited, this proof of concept provides an interesting approach to estimate the second-by-second dynamics of mental states beyond the classical minute-by-minute sampling of subjective experience, paving the way for a fine-grained exploration of the dynamics of consciousness, without undersampling or interfering with these dynamics by requiring a verbal report. Incorporating more detailed descriptions of mental states or embracing a multidimensional description of subjective experience could enhance both the accuracy and generalizability of such classifiers across individuals or groups, as recently shown in the context of fMRI studies (11, 91).

### Conclusion: Is MB a State without Conscious Content During Wakefulness?

In the introduction of this paper, we presented the predicted neural correlates of a potential contentless conscious state during wakefulness, based on theoretical (33, 34, 92) and empirical (30, 38, 44, 45, 67) considerations. We showed here that the state of MB, a phenomenological, behavioral, and neurophysiological distinct state from MW, fulfills these predictions. First, as predicted, the sharing of information between distant cortical areas was disrupted during MB, in particular in the delta and theta frequency bands. Second, the neural representations of external stimuli were significantly disrupted during MB, starting from early periods of processing and echoing into late processing, usually associated with conscious access, with a lack of the usual signatures of conscious access during this mind state. Finally, spectral, complexity, and coherence based connectivity metrics point toward the hypothesis of neural silencing of posterior associative cortices, a key node for consciousness according to most theoretical accounts (34, 93). Since we studied MB in a specific context and task-design, it is possible that some of the markers obtained here would not generalize to other instances of MB. Yet, some of the markers evidenced in this study are compatible with those obtained using a different neuroimaging technique (fMRI) and without a task (21, 23).

The presence or absence of internally generated representations during this mind state remains an open question, and the impression of an “empty mind” reported by the participants during MB could be accounted for by different mechanisms (i.e., lack of metacognitive awareness, lack of memory encoding, language limitations) other than a lack of conscious experience altogether (19). Still, based on the above presented results, we argue in favor of the more radical interpretation of MB as a “content-free” mind state. This would challenge our intuition of a continuous conscious content during wakefulness. In this view, conscious experience would be a discrete phenomenon, with discrete temporal islets with conscious content, separated by brief contentless periods (92). While puzzling, this perspective would bring even more value to those precious moments of conscious experience.

### Online Methods.

Sixty-eight healthy adults participated in a modified visual SART, conducted over approximately 100 min, during which high-density EEG and oculometric data were continuously recorded. At various points during the task, participants were interrupted and asked to report their current mental state: specifically, whether they were focused on the task (task-focused), thinking about something unrelated to the task (MW), or not thinking about anything in particular (MB). We then compared behavioral and EEG data preceding these interruptions across the three reported mental states. A detailed account of the experimental procedures and data analyses is provided in *SI Appendix*, Supplementary Materials.

## Supplementary Material

Appendix 01 (PDF)

## Data Availability

EEG and behavioral data have been deposited in Zenodo (https://doi.org/10.5281/zenodo.17649277) (94). Previously published data were used for this work (https://osf.io/ey3ca/) (95).
